# Monte Carlo characterization of ${}^{169}\text{Yb}$ as a high‐dose‐rate source for brachytherapy application by FLUKA code

**DOI:** 10.1120/jacmp.v14i4.4298

**Published:** 2013-07-08

**Authors:** Marzieh Anjomrouz, Mahdi Sadeghi, Asghar Haddadi

**Affiliations:** ^1^ Medical Radiation Engineering Department Science and Research Branch, Islamic Azad University Tehran Iran; ^2^ Agricultural Medical and Industrial Research School, Nuclear Science and Technology Research Institute Karaj Iran

**Keywords:** brachytherapy, high‐dose‐rate source, ${}^{169}\text{Yb}$, Monte Carlo, FLUKA

## Abstract

Higher initial dose rate and simplifying HDR room treatment of ${}^{169}\text{Yb}$ element among other brachytherapy sources has led to investigating its feasibility as high‐dose‐rate seed. In this work, Monte Carlo calculation was performed to obtain dosimetric parameters of ${}^{169}\text{Yb}$, Model M42 source at different radial distances according to AAPM TG‐43U1 and HEBD Report about HDR sources in both air vacuum and spherical homogeneous water phantom. The deposited energy resulted by FLUKA as Monte Carlo code using binning estimators around ${}^{169}\text{Yb}$ source was converted into radial dose rate distribution in polar coordinates surrounding the brachytherapy source. The results indicate a dose rate constant of $1.14\pm 0.04\;\text{cGy}.h^{-1}.U^{-1}$ with approximate uncertainty of 0.04%, air kerma strength, $1.082\pm 2.6E-06\;U.{\text{mCi}}^{-1}$ and anisotropy function ranging from 0.386 to 1.00 for radial distances of 0.5–10 cm and polar angles of 0°–180°. Overall, FLUKA dosimetric outputs were benchmarked with those published by Cazeca et al. via MCNP5 as one of validate dosimetry datasets related to ${}^{169}\text{Yb}$ HDR source. As a result, it seems that FLUKA code can be applicable as a valuable tool to Monte Carlo evaluation of novel HDR brachytherapy sources.

PACS number: 87.15.ak

## I. INTRODUCTION

Radionuclides of lanthanide group are gaining importance as emerging therapeutic agents in nuclear medicine and radiation therapy.^1^, ^2^ ${}^{169}\text{Yb}$ is a lanthanide element that can be produced by neutron activation of ${}^{168}\text{Yb}$ in a nuclear reactor.^(3)^ ${}^{169}\text{Yb}$ with a half‐life of 32.02±0.009 days decays by electron capture to ${}^{169}\text{Tm}$ and emits a low‐energy photon spectrum with average energy of 93 keV.^4^, ^5^ It has an equivalent dose distribution similar to ${}^{192}\text{Ir}$ in water, but a much smaller half‐value layer of 0.2 mm in lead and average tenth value thickness of 1 mm.^(4)^ Therefore, these valuable features result in improved radiation protection consisting of simplified HDR room treatment with low level of installation costs, customized shielding of dose‐limiting anatomic structures, and fabrication of shielded applicator to modulate the desirable dose distribution to achieve the specific requirements during adaptive brachytherapy treatment planning.^(4)^ Moreover, it has a slightly higher initial dose rate of $12.5\;\text{cGy}.h^{-1}$, thus self‐attenuation of medium, particularly soft tissue, would be negligible in comparison with other high‐dose‐rate (HDR) sources,^(6)^ and penetration power would help to candidate ${}^{169}\text{Yb}$ as a valuable HDR sources in various treatment techniques of tumors such as intravascular brachytherapy (IVBT), prostate, breast, tongue, and gynecological applications.^4^, ^7^, ^8^

According to the American Association of Physicists in Medicine (AAPM) TG‐43U1 recommendations, before using each new source in treatment planning calculations, the dosimetric characteristics of the source must be determined to provide reliable data.^9^, ^10^, ^11^, ^12^ The dosimetry dataset of ${}^{169}\text{Yb}$ as a novel HDR source has not been implemented to new updated reports and it has not been commercially fabricated yet. Therefore various models of ${}^{169}\text{Yb}$ have been investigated by experts, using different dosimetry characterization methods, ranging from Monte Carlo calculations to experimental measurements, with various level of perception of their results.^(11)^ For instance, Cazeca et al.^(5)^ evaluated dosimetry parameter based on Monte Carlo method like MCNP5, and Chiu‐Tsao et al.^(3)^ in 2000 investigated ${}^{169}\text{Yb}$ application on IVBT and presented results on the intravascular brachytherapy. In 2013, a paper was published by Saidi et al.^(13)^ concerning experimental measurement and Monte Carlo methods of a type of eye plaques loaded with IRA1‐${}^{103}\text{Pd}$ seeds for the radiation therapy application of choroidal melanoma. In some studies, authors compared different sources with each other to measure dose distribution and select appropriate source to special application radiotherapy. Mainegra and Capotez^(14)^ in 1997 compare dose rate constants for ${}^{125}I$, ${}^{103}\text{Pd}$, ${}^{192}\text{Ir}$, and ${}^{169}\text{Yb}$ brachytherapy sources via EGS4 as a Monte Carlo code. In 2005, a dosimetric comparison of ${}^{169}\text{Yb}$ versus ${}^{192}\text{Ir}$ for HDR prostate brachytherapy was done by Lymperopoulou et al.^(15)^ and according to their results, the shielding requirements for the ${}^{169}\text{Yb}$ energies are minimal relative to those for ${}^{192}\text{Ir}$. Furthermore, in some studies various techniques have been reviewed in order to reach dosimetric fundamentals for routine clinical use of HDR photon emitting brachytherapy sources, such as a publication by Li et al.^(16)^ in 2007. In the current study ${}^{169}\text{Yb}$ source, Model M42, has been simulated via FLUKA as a Monte Carlo method and TG‐43U1 brachytherapy dosimetry parameters such as anisotropy function, radial dose function, air kerma strength, and dose‐rate constant have been obtained. Ultimately, to validate the data resulted by FLUKA, they have been compared with the results of similar source model as was used by Cazeca et al. based on MCNP5 scores.

## II. MATERIALS AND METHODS

### A. ${}^{169}\text{Yb}$ source description

**Figure 1:**
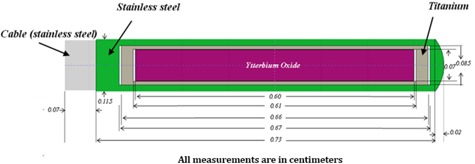
Schematic diagram of ${}^{169}\text{Yb}$ HDR source, Model M42.

The source model employed in this research is similar to that applied in the investigation performed by Cazeca et al.^(5)^ for the Monte Carlo analysis. The ${}^{169}\text{Yb}$ HDR source, Model M42, consists of a cylinder loaded by Ytterbium oxide of $7.1\;\text{mg}.{\text{mm}}^{-3}$ and encapsulated by two long cylinders. The inner cylinder is made of Titanium of $4.51\;\text{mg}.{\text{mm}}^{-3}$ and outer one is made of 304 stainless steel of $7.80\;\text{mg}.{\text{mm}}^{-3}$ composition of 16% Cr, 10% Ni, 0.03% S, 0.75% Si, 0.08% C, 2% Mn, 0.045% P, 69.095% Fe, and 2% Mo. This source placed at the center of a 50 cm radius spherical phantom filled with homogenous water. The schematic view of ${}^{169}\text{Yb}$, Model M42, is presented in Fig. 1 along with the coordinate system used. Due to the addition of an attached cable, the source assembly is asymmetric about the transverse plane principally but symmetric about the coaxial axis.

### B. Monte Carlo calculation method

In order to simulate photon transport of hypothetical ${}^{169}\text{Yb}$ HDR source situated once in center of a water phantom and in the air vacuum another time. To extract proportional scores, version 2011.2.14 (updated in 17th October 2012) of FLUKA code was utilized in the present research. This code is a multipurpose Monte Carlo code which has been developed for high accurate simulation the interaction and propagation in matter of about 60 different particles, including photons and electrons from 1 keV to thousands of TeV, neutrinos, muons of any energy, hadrons of energies up to 20 TeV and all the corresponding antiparticles, neutrons down to thermal energies and heavy ions. Moreover, time evolution and tracking of emitted radiation from unstable residual nuclei can be performed online by this code. Decay scoring, one of the valuable capabilities of this code, has been carried out in this study.^(17–20^)

#### B.1 Essential setting

The lanthanide source of ${}^{169}\text{Yb}$ emits a photon spectrum that could be defined in the source routine and then it should be attached to FLUKA input. In the recent releases, new particle name has been implemented to beam card called ISOTOPE. By choosing this option and determining the type of isotope via the HI‐PROBE card, the code applies relevant spectrum to input file, conveniently.

Since, in the current research, radioisotope decay is considered as a radiation source; therefore, appropriate cards of decay such as RADDECAY should be applied in the transport section of Flair as a FLUKA GUI. Furthermore, the card of DCYSCORE should be embedded in the scoring setup and then linked to the dose estimator of USRBIN.

In addition, the type of interaction, as well as the adequate thresholds, can be set in the physic and transport sections and to enable pure electromagnetic intersection input file was carried out in electromagnetic FLUKA (EMF) cascade mode.^(17)^ Finally, this code was set at $3\times 10^{8}$ of initial photon histories to minimize relative statistical error of simulation which varied from $3\times 10^{-4}\%$ to $8.31\times 10^{-1}\%$ with decreasing distance from source.

#### B.2 Geometry definition

**Figure 2:**
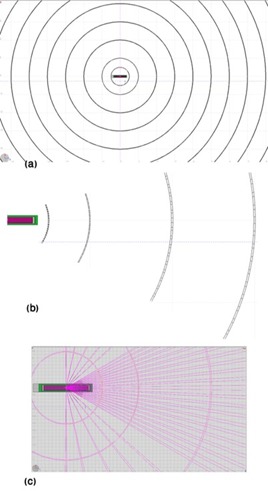
A cross section of the detector arrangement around ${}^{169}\text{Yb}$ source, Model M42, designed by FLUKA geometry to obtain in‐phantom anisotropy and radial dose information in various scales: (a) overview of seed and ring detectors; (b) source and detectors in larger scale; (c) intersection of cones and spherical shells’ bodies.

According to common layout of detectors in the literature,^(5^) the group of detectors is usually composed of ring‐shaped array formed by a chain of concentric spherical shells intersected by a chain of concentric cones. Figure 2 shows the detector arrangement defined by FLUKA geometry, in which 0.5 cm thickness of shells in 1 cm increments from 1 cm to 10 cm and angular aperture of cones in steps of 3° from 2° to 89°.

**Figure 3:**
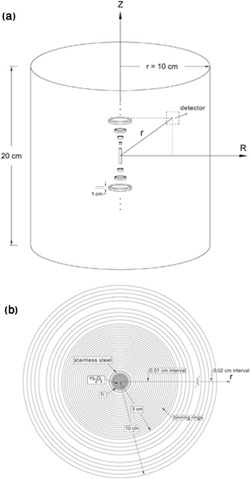
Configuration of region binning by FLUKA geometry: (a) the view of virtual cylindrical rings; (b) cross‐sectional view of sources along with ring detectors.

In spite of MCNP code, the number of bodies and regions in FLUKA does not have low limitation for geometry definition, which of course was not utilized in current work because of increasing run‐time and decreasing simulating accuracy.^(17)^ Thus, use of region binning method would be suggested. In fact, this novel approach was performed by definition of useful virtual cylindrical binning on the geometry in USRBIN cards in structure of the mesh R‐Phi‐Z (Fig. 3) as a deposited energy estimator in FLUKA code in unit of GeV per source particle and air‐kerma strength were calculated in separate runs, in different structure.

Ultimately, dataset delivered by FLUKA estimators has been selected by replacement of the calculated position of detectors in phantom of water. To obtain the dose in various detectors, output results were divided by volume of each virtual bin, and then selected data were converted to $\text{cGy}.h^{-1}.{\text{mCi}}^{-1}$.

It should be noted that various data between two or four common virtual surfaces should be averaged. The input file has been run by a personal computer with Dual‐Core CPU and Linux as an operating system; as a result, radiation dose in given detectors would be accessible with average error of well under $2.5\times 10^{-2}\%$.

#### B.3 Obtaining dosimetry dataset

In the most relevant literatures, updated AAPM Task group 43 (TG‐43U1) recommendations have been referred to evaluate high‐dose‐rate sources. In August 2012, AAPM and ESTRO‘s working group of High Energy Brachytherapy source Dosimetry (HEBD) issued a separate full report focused on dose calculation for photon‐emitting brachytherapy sources with average energy higher than 50 keV.^(21)^ As claimed by this report, the TG‐43U1 guidelines and recommendations are applicable to HDR sources, unless configurations of sources are specified in new report based on some characteristics such as active length of source.

According to both standard methodologies with respect to cylindrical sources, the proposed formula for two‐dimensional dose rate is:^11^, ^21^

(1) $$\dot{D}(r,\theta )=S_{K}.\Lambda .\frac{G_{L}(r,\theta )}{G_{L}(r_{0},\theta_{0})}.g_{L}(r).F(r,\theta )$$

In this equation, dose rate is accessible via a given FLUKA estimator to evaluate absorbed dose distribution which utilized in other parameters such as dose rate constant, Λ, was calculated by dividing the dose rate, $\dot{D}(r,\theta )$, at 1 cm in water by the calculated air‐kerma rate, ${\dot{K}}_{\delta }(d)$, at 1 m in air.^11^, ^21^ Due to photon collisions, the produced electrons are low energy, and they are absorbed locally without any scattering or absorption in dosimetry measurement point,^(22)^ and it could be assumed that dose is equal to kerma at the demand point. The air‐kerma rate has been calculated by separate input file in which its geometry configured in a 1 m radius vacuum phantom surrounded by an air‐filled ring detector made by an intersection of two spherical shells with inner and outer radius of 97.5 and 102.5, respectively, and two cones with 88° and 92° angles of aperture.^(5)^ Consequently, by 1×108 histories, air‐kerma rate was estimated $1.981\times 10^{-4}\;\text{cGy}.h^{-1}.{\text{mCi}}^{-1}$ with statistical uncertainty of about $2.63\times 10^{-6}\%$. Air‐kerma strength ($S_{k}$) would be obtained by replacement of air‐kerma rate in various positioned detectors based on distance, (Eq. (2)):^11^, ^21^

(2) $$S_{k}={\dot{K}}_{\delta }(d)d^{2}$$

Obviously, air kerma rate follows inverse square law, owing to attenuation and scattering of photons in air. Moreover other dosimetric characteristics, including geometry function, $G_{L}$, radial dose function ($g_{L}(r)$), and anisotropy function (F(r,Θ)), were calculated in similar approach to that described in TG‐43U1 protocol and HEBD report.

## III. RESULTS & DISCUSSION

The procedure applied to obtain dosimetry dataset for ${}^{169}\text{Yb}$ source, Model M42, in current issue has been evaluated by simulation of similar source characterized by Cazeca et al.^(5)^ with MCNP5 and could be applied as an appropriate benchmark of obtained results.

**Figure 4:**
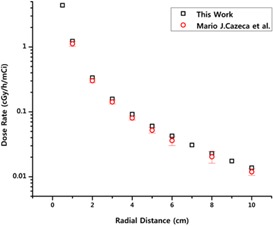
The figure illustrates a comparison between dose rate of current study and data generated by Cazeca et al.^(5)^ in various distances up to 10 cm and polar angle 90°.

As mentioned in previous section, scoring data from FLUKA estimators in water phantom were converted to dose rate, $\dot{D}(r,\theta )$, at the radial distance, r, and the polar angle, Θ, and have been presented at Table 1. The results of dose rate of polar angle 90° in various distances up to 10 cm are illustrated in Fig. 4, in comparison to reference data. Clearly, it could be seen that a complete conformity between two datasets of current research and the Cazeca study.

**Table 1:** Dose rate in water ($\text{cGy}.H^{-1}.{\text{mCi}}^{-1}$) calculated by FLUKA. The source is oriented along the z‐axis with the origin at the source center

	*r (cm)*										
θ(deg)	*0.5*	*1*	*2*	*3*	*4*	*5*	*6*	*7*	*8*	*9*	*10*
0	4.544	0.968	0.199	0.084	0.063	0.036	0.029	0.023	0.017	0.015	0.010
10	4.353	0.808	0.212	0.108	0.055	0.038	0.032	0.024	0.017	0.013	0.011
20	5.013	0.996	0.290	0.132	0.071	0.047	0.032	0.025	0.018	0.014	0.011
30	4.905	1.129	0.294	0.133	0.080	0.047	0.038	0.027	0.020	0.015	0.011
40	5.000	1.184	0.304	0.152	0.081	0.053	0.038	0.027	0.021	0.015	0.012
50	4.881	1.216	0.323	0.150	0.085	0.054	0.037	0.027	0.020	0.016	0.012
60	4.559	1.225	0.326	0.151	0.088	0.057	0.039	0.029	0.021	0.016	0.013
70	4.462	1.238	0.337	0.160	0.090	0.058	0.041	0.029	0.021	0.016	0.013
80	4.387	1.241	0.334	0.159	0.092	0.060	0.041	0.029	0.022	0.017	0.014
90	4.399	1.229	0.336	0.158	0.092	0.060	0.042	0.030	0.022	0.017	0.013
100	4.324	1.229	0.341	0.158	0.091	0.057	0.042	0.030	0.022	0.016	0.013
110	4.465	1.241	0.331	0.156	0.088	0.057	0.040	0.028	0.021	0.016	0.013
120	4.607	1.217	0.320	0.158	0.088	0.057	0.039	0.028	0.021	0.017	0.013
130	4.844	1.227	0.326	0.148	0.086	0.054	0.039	0.027	0.020	0.017	0.012
140	5.100	1.167	0.311	0.144	0.084	0.051	0.038	0.028	0.020	0.015	0.012
150	4.977	1.098	0.287	0.135	0.076	0.050	0.036	0.026	0.019	0.015	0.012
160	5.018	0.945	0.271	0.120	0.073	0.047	0.032	0.025	0.017	0.013	0.011
170	3.902	0.754	0.220	0.100	0.057	0.045	0.029	0.020	0.017	0.013	0.010
180	3.748	0.943	0.133	0.100	0.061	0.040	0.026	0.022	0.016	0.012	0.010

The radial dose function, $g_{L}(r)$, was achieved by using dose rate parameter based on standard formalism ^11^, ^21^ for ${}^{169}\text{Yb}$ HDR source with active length, L, of 0.6 cm.

**Figure 5:**
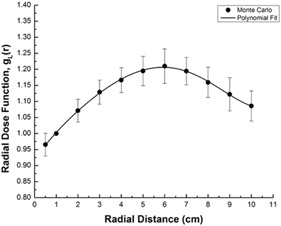
Radial dose function, $g_{L}(r)$ and the fifth order polynomial function fit.

The difference between the two studies is tabulated in Table 2. This difference could be related to variety of cross‐sectional data utilized by FLUKA and MCNP5. Furthermore, by using Eq. (3), this radial dose function was fitted to a fifth order polynomial for commercial treatment planning purpose (Fig. 5):

(3) $$g_{L}(r)=a_{0}+a_{1}r+a_{2}r^{2}+a_{3}r^{3}+a_{4}r^{4}+a_{5}r^{5}$$

Where $a_{0}=9.19\times 10^{-1},a_{1}=9.52\times 10^{-2},a_{2}=-1.39\times 10^{-2},a_{3}=2.77\times 10^{-3},a_{4}=-4.12\times 10^{-4}$, and $a_{5}=1.96\times 10^{-5}$, and define $R^{2}=0.999$.

The 2D anisotropy function, F(r,Θ), of ${}^{169}\text{Yb}$ source, as another dosimetry parameter can be calculated by using of dose rate by means of FLUKA simulation, is reported in Table 3 in various radial distance, r, from 0.5 to 10 cm relative to source center, and polar angle, Θ, from 0° to 180° with respect to the source long axis. In fact, this factor indicates various doses in the longitudinal plane of source, because of different distribution of radioactivity along with cylindrical source, self‐absorption, and self‐filtration of seed layers.^(22)^

**Figure 6:**
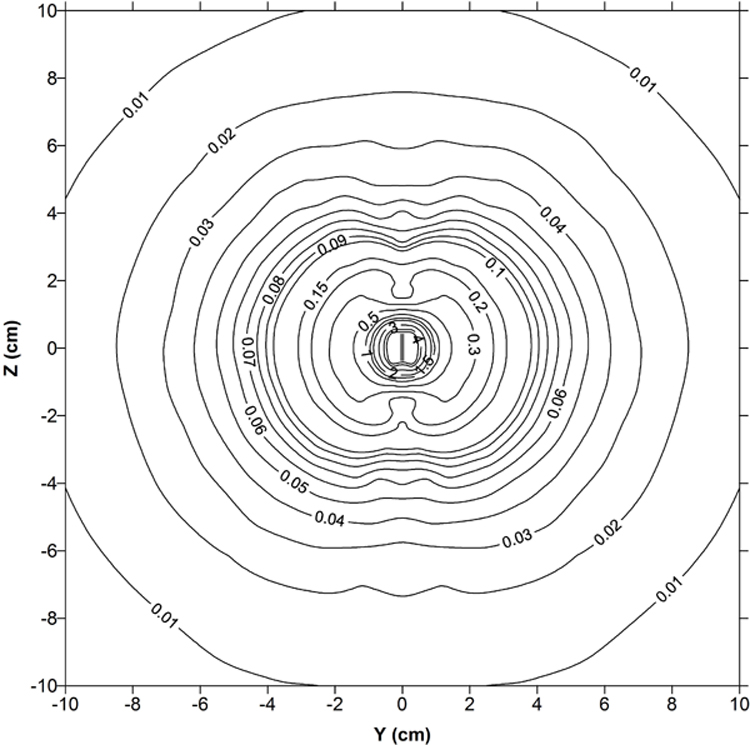
Calculated isodose curves around ${}^{169}\text{Yb}$, Model M42.

Besides, the elliptical isodose curves in Fig. 6 demonstrate dose distribution around source. The significant dose attenuation can be seen in both end caps which applied in treatment planning purposes.

**Table 2:** Radial dose function, $g_{L}(r)$

	$g_{L}(r)$		
*r(cm)*	*FLUKA*	*MCNP5*	*Difference*
0.5	0.965	0.945	2.07%
1	1.000	1.000	0.00%
2	1.071	1.081	−0.93%
3	1.129	1.131	−0.18%
4	1.167	1.158	0.77%
5	1.195	1.168	2.26%
6	1.210	1.165	3.72%
7	1.194	1.151	3.60%
8	1.159	1.128	2.67%
9	1.122	1.098	2.14%
10	1.086	1.062	2.21%

**Table 3:** Anisotropy function F(r,Θ), calculated by FLUKA code

	*r (cm)*										
θ(deg)	*0.5*	*1*	*2*	*3*	*4*	*5*	*6*	*7*	*8*	*9*	*10*
0	0.595	0.696	0.574	0.526	0.688	0.605	0.692	0.765	0.778	0.860	0.770
10	0.590	0.584	0.612	0.680	0.601	0.634	0.750	0.802	0.747	0.764	0.842
20	0.738	0.728	0.841	0.825	0.768	0.775	0.765	0.837	0.811	0.841	0.852
30	0.800	0.840	0.857	0.836	0.874	0.785	0.897	0.875	0.893	0.875	0.863
40	0.899	0.900	0.889	0.959	0.883	0.886	0.913	0.897	0.939	0.883	0.942
50	0.954	0.945	0.951	0.947	0.927	0.905	0.891	0.902	0.900	0.960	0.924
60	0.952	0.970	0.965	0.952	0.954	0.952	0.934	0.966	0.937	0.934	0.983
70	0.976	0.995	0.999	1.011	0.977	0.961	0.963	0.962	0.956	0.943	1.011
80	0.987	1.006	0.992	1.008	1.007	1.000	0.985	0.946	0.964	1.008	1.021
90	1.000	1.000	1.000	1.000	1.000	1.000	1.000	1.000	1.000	1.000	1.000
100	0.973	0.997	1.015	1.005	0.991	0.953	0.987	0.994	0.967	0.967	0.961
110	0.977	0.997	0.981	0.986	0.960	0.952	0.959	0.934	0.954	0.949	0.975
120	0.962	0.963	0.945	0.996	0.958	0.956	0.925	0.939	0.949	0.978	0.972
130	0.947	0.953	0.960	0.932	0.933	0.903	0.918	0.898	0.916	0.979	0.934
140	0.917	0.888	0.910	0.904	0.913	0.853	0.899	0.923	0.879	0.911	0.891
150	0.811	0.818	0.834	0.847	0.825	0.839	0.846	0.845	0.857	0.889	0.939
160	0.739	0.691	0.784	0.756	0.789	0.781	0.764	0.832	0.776	0.777	0.859
170	0.528	0.544	0.637	0.626	0.620	0.743	0.693	0.662	0.761	0.781	0.788
180	0.491	0.678	0.385	0.627	0.667	0.663	0.630	0.733	0.698	0.737	0.754

The aforementioned air‐kerma strength, $S_{k}$, was calculated as $1.082\pm 2.6E-06\;U.{\text{mCi}}^{-1}(1U=1\mu {\text{Gy m}}^{2}\;h^{-1})$, and is in good agreement with the study by Cazeca and colleagues of $1.08\pm 0.03\;U.{\text{mCi}}^{-1}$. The dose rate constant value, Λ, as accuracy criteria of present Monte Carlo simulation in comparison with reference work was obtained by $1.14\pm 0.04\;\text{cGy}.h^{-1}.U^{-1}$, which is close to $1.12\pm 0.04\;\text{cGy}.h^{1}.U^{-1}$ found by Cazeca et al.^(5^)

## IV. CONCLUSIONS

The dosimetry of ${}^{169}\text{Yb}$ HDR brachytherapy source, Model M42, was investigated in this study in association with FLUKA estimators, according to AAPM standard methodology. In summary, simulation results of FLUKA code are consistent with those published by Cazeca et al.^(5)^ via MCNP5 as one of validate dosimetry datasets related to ${}^{169}\text{Yb}$ HDR source. Actually, it seems that FLUKA code with high quality of GUI can be facilitated by implementing standard protocols’ procedures similar to MCNP. Therefore, it can be applicable as a valuable alternative tool to Monte Carlo evaluation of novel HDR brachytherapy sources.

## ACKNOWLEDGMENTS

The authors would like to thank Dr. Pooneh Saidi for her assistance in editing this paper.
